# Human umbilical cord mesenchymal stem cells ameliorate colon inflammation via modulation of gut microbiota-SCFAs-immune axis

**DOI:** 10.1186/s13287-023-03471-9

**Published:** 2023-09-25

**Authors:** Airu Liu, Xiaonan Liang, Wenxin Wang, Chen Wang, Jia Song, Jinbo Guo, Donglei Sun, Dong Wang, Mei Song, Jiaming Qian, Xiaolan Zhang

**Affiliations:** 1 Hebei Key Laboratory of Gastroenterology, Hebei Institute of Gastroenterology, Hebei Clinical Research Center for Digestive Diseases, Department of Gastroenterology, The Second Hospital of Hebei Medical University, Shijiazhuang City, China; 2 Department of Gastroenterology, Peking Union Medical College Hospital, Chinese Academy of Medical Sciences and Peking Union Medical College, Beijing City, China

**Keywords:** Mesenchymal stem cells (MSCs), Colitis, Gut microbiota, Short-chain fatty acids (SCFAs), CD4^+^T homeostasis

## Abstract

**Background:**

Inflammatory bowel disease (IBD) is a global health problem in which gut microbiota dysbiosis plays a pivotal pathogenic role. Mesenchymal stem cells (MSCs) therapy has emerged as a prospective novel tool for managing IBD, and which can also regulate the composition of gut microbiota. However, the functional significance of MSCs-induced changes in gut microbiome is poorly understood.

**Methods:**

Here, we investigated for the first time the role of gut microbiota in mediating the protective effect of human umbilical cord MSCs (HUMSCs) on DSS-induced colitis. Gut microbiota alteration and short-chain fatty acids (SCFAs) production were analyzed through 16S rRNA sequencing and targeted metabolomics. Spectrum antibiotic cocktail (ABX), fecal microbiota transplantation (FMT) and sterile fecal filtrate (SFF) were employed to evaluate the protective effect of intestinal flora and its metabolites. Cytokine microarray, Enzyme-linked immunosorbent assay (ELISA), and flow cytometry were conducted to assess the effect on CD4^+^T homeostasis.

**Results:**

Here, we investigated for the first time the role of gut microbiota in mediating the protective effect of MSCs on DSS-induced colitis. By performing gut microbiota depletion and fecal microbiota transplantation (FMT) experiments, we revealed that MSCs derived from human umbilical cord ameliorated colon inflammation and reshaped T-cells immune homeostasis via remodeling the composition and diversity of gut flora, especially up-regulated SCFAs-producing bacterial abundance, such as *Akkermansia*, *Faecalibaculum*, and *Clostridia_UCG_014*. Consistently, targeted metabolomics manifested the increased SCFAs production with MSCs administration, and there was also a significant positive correlation between differential bacteria and SCFAs. Meanwhile, combined with sterile fecal filtrate (SFF) gavage experiments, the underlying protective mechanism was further associated with the improved Treg/Th2/Th17 balance in intestinal mucosa mediated via the increased microbiota-derived SCFAs production.

**Conclusion:**

The present study advances understanding of MSCs in the protective effects on colitis, providing evidence for the new role of the microbiome-metabolite-immune axis in the recovery of colitis by MSCs.

**Supplementary Information:**

The online version contains supplementary material available at 10.1186/s13287-023-03471-9.

## Background

Inflammatory bowel diseases (IBD), including Crohn’s disease (CD) and ulcerative colitis (UC), are chronic idiopathic disorders characterized by non-specific recurrent intestinal inflammation [1]. IBD has emerged as a global health challenge with increasing morbidity, decreased quality of life, and colitis-associated colorectal cancer (CAC) progression [2]. Despite the emergence of new biologics and small molecule drugs, their therapeutic effects are not satisfactory, and long-term use of these drugs still increases the risk of adverse events such as serious infections, neurological diseases, malignancies, and thrombosis [3]. Therefore, alternative safe and effective treatments need to be developed.

Although the pathogenesis of IBD has not yet been elucidated, it is generally believed that intestinal microbial imbalance as the trigger point of IBD event derails immune homeostasis and touches off immune-mediated intestinal mucosal inflammatory response [4]. CD4^+^ T cells are the main effector cells of intestinal mucosal inflammatory response. Naïve CD4^+^ T cells can be induced to differentiate into regulatory T (Treg) cells and T helper (Th) cells under antigen stimulation [5]. Treg cells perform immunosuppressive function to inhibit the activity of pro-inflammatory Th1 and Th17 cells by secreting IL-10, decreasing the secretion of IFN-γ, IL-17A, thereby maintaining host immune homeostasis, and Th2 cells can also show a synergistic effect with Treg cells by secreting inhibitory cytokine, such as IL-4 and IL-10 [6, 7]. However, intestinal dysbiosis is considered the critical factor to break this homeostasis and promote the occurrence of IBD. Enormous evidence has unveiled the presence of microbial imbalance in IBD patients, marked by decreased microbial diversity and altered flora, including decrease in certain genera of the phylum *Bacteriodetes* and *Firmicutes*, and increased level of *Actinobacteria* and *Proteobacteria* [4]. Transfer fecal microbiota from IBD patients to germ-free mice induced colitis through perturbing the balance of Treg/Th2/Th17 [8], and while transplanting fecal flora from healthy people to mice with experimental colitis significantly improved the immune-inflammatory state of mice, reshaping Th1/Th17/Treg balance, downregulating the level of pro-inflammatory cytokines, including TNF-α, IFN-γ, IL-1β and increasing the level of IL-10 [9]. Although the mechanism by which the gut microbiota regulates CD4^+^ T cells remains unclear, Multitudes of studies have elucidated that short-chain fatty acids (SCFAs) as key metabolites derived from gut microbiota, participate in the maintenance of intestinal homeostasis as well as in the pathogenesis of IBD [10]. The principal SCFAs, including acetate, propionate and butyrate, especially butyrate, have been confirmed to suppress the proliferation activity of CD4^+^ T cells, significantly promote Treg pool in the colonic lamina propria, and upregulate the secretion of IL-10, improving colon inflammation [11, 12]. Collectively, a close regulatory relationship exists between intestinal microecology and intestinal mucosal immunity, it is of great value and practical significance to improve the clinical efficacy of IBD via correcting the intestinal dysbiosis.

Mesenchymal stem cells (MSCs) therapy, as a novel strategy, has shown promising efficacy and safety in IBD field mainly due to its powerful immunoregulation and the ability of tissue regeneration [13, 14]. Immunologically, MSCs delivery regulates intestinal immune homeostasis, especially CD4^+^T cell adaptive immune response, reshaping Treg/Th17 balance and inhibiting the secretion of pro-inflammatory cytokine [13]. While the latest studies revealed that MSCs also remodeled the diversity and structure of gut flora with similar composition of bacterial taxa to that of normal mice [15, 16], and meanwhile increased the levels of SCFAs-producing bacteria*,* such as *Akkermansia*, *Clostridium* and *Lachnospiraceae* [17, 18]. In addition, a study pointed that MSCs therapy could correct intestinal dysbiosis in mice with chronic colitis, clearly decreasing the abundance of *Bilophila and E. brachy* closely related to colorectal cancer, which may be a potential therapeutic mechanism for MSCs to reduce the risk of CAC [19]. Generally, MSCs can also remodel the composition and diversity of gut microbiota, while the functional significance of MSCs-induced changes in gut microbiome is not yet elucidated.

In the current study, we investigated for the first time the role of gut microbiota in mediating the protective effect of MSCs on DSS-induced colitis. Gut microbiota depletion, FMT and sterile fecal filtrate (SFF) gavage experiments were conducted. Finally, the microbiota-metabolic-immune axis by which MSCs improved colonic inflammation was explored.

## Materials and methods

### Cell preparation and culture

Human umbilical cord mesenchymal stem cells (HUMSCs) purchased from Cyagen Biosciences (Guangzhou, China) were cultured in a MSC medium (OriCell®, Cyagen Biosciences, Guangzhou, China) at 37 °C in a 5% CO2 incubator. Flow cytometry was applied to verify the phenotype of HUMSC through examining the expression of cell surface markers, such as positive markers CD44, CD105, CD90, CD73, CD29 and negative markers HLA-DR and CD45. HUMSCs from passages 3 and 6 were used throughout the experiments.

### Animals

Seven- to eight-week-old wild-type specific pathogen-free (SPF) C57BL/6 male mice (weight, 20–23 g) (Beijing Vital River Laboratory Animal Technology Co. Ltd) were used to construct colitis models. The mice were housed in a SPF animal laboratory with a constant temperature of 22 °C (± 2 °C) and a fixed 12-h light–dark cycle. All animal experimental protocols were performed following the guidelines of the National Institutes of Health guide for the care and use of Laboratory Animals, and approved by the Research Ethics Committee of the second hospital of Hebei Medical University. The reporting of animal experiments adheres to the ARRIVE guidelines (https://arriveguidelines.org/arrive-guidelines).

### DSS-induced colitis

The mice were randomly divided into three groups following the random number table. As we previously reported [20], colitis was induced in mice by oral delivery of 2% dextran sodium sulfate (DSS) (MP Biomedicals, USA) in drinking water ad libitum for 7 days followed by 3 days normal water. MSCs (1 × 10^6^ cells) suspended in 200 μL phosphate-buffered saline (PBS) were administered by intraperitoneal (i.p.) injection in DSS/MSC group mice on day 5 of the study (with DSS administration initiated on day 0). While the Control and DSS groups of mice were administered with the same volume of PBS by i.p. injection [21, 22]. To avoid abdominal infection caused by i.p. injection, the procedures were strictly performed under sterile conditions. At the end of treatments, mice were euthanized by cervical dislocation following 1.5% isoflurane inhalation anesthesia in an anesthesia induction chamber. Deaths of mice not associated with experimental intervention would be excluded.

### Assessment of colitis

In all colitis models, mice were checked daily for body weight and survival. Clinical features, including body weight loss, presence of blood stool, and stool consistency were combined to generate the Disease Activity Index (DAI) evaluating disease severity as we previously described [20]. The mice were sacrificed after anesthesia on day 10, the length of the colon was measured. The dissected colon tissues were fixed in 4% formalin for 48 h and then embedded into paraffin. 4-μm slides were stained with hematoxylin and eosin (H&E) for histopathological analysis to assess the colon damage severity by Cooper HS score system [20].

### Antibiotic treatment of mice

Mice were treated with a broad-spectrum cocktail of antibiotics (ABX: vancomycin, 100 mg/kg; neomycin sulfate 200 mg/kg; metronidazole 200 mg/kg; and ampicillin 200 mg/kg) intragastrically once a day for 5 days to deplete gut microbiota as described previously [23], and then 2% DSS drinking water was administered for 7 days to induce colitis. ABX/DSS/MSC group mice were treated with 1 × 10^6^ HUMSCs (i.p.), and ABX/DSS/PBS and ABX/Control group mice were delivered an equal volume of vehicle in the same way.

### FMT and SFF

The frozen feces from donor mice (Control, DSS/PBS and DSS/MSC groups) were pooled and homogenized in sterile PBS with a final concentration of 100 mg feces/mL under anaerobic conditions. Samples were vortexed (2 min), centrifuged (5 min at 600 g) and 200 μL of the fecal supernatant was given to ABX-mice by oral gavage once a day for continuous 5 days to perform FMT [24]. In other experiments, the fecal supernatant was collected and centrifuged (5 min at 12,000×*g*) and then passed through 0.22 μm filters to obtain SFF [25]. 400 μL of the sterile filtrate was then administered orally to ABX-mice once a day for continuous 10 days.

### SCFAs administration in mice

For investigating the effect of SCFAs in protecting mice from DSS-colitis, ABX-mice were intragastrically delivered with a mixture of 400 µL SCFAs [1% (w/v) dissolved in water] including 99.5% sodium acetate (C14014780; Macklin), 99.0% sodium propionate (C13860066; Macklin) and 98.0% sodium butyrate (C13817958; Macklin) with a ratio of 4:3:3 for continuous 10 days.

### 16S rRNA sequencing

The fecal samples for 16S rRNA sequencing analysis were pooled and stored at − 80 °C. The fecal genome DNA was extracted using CTAB/SDS method as previously described [26]. DNA concentration and quality were detected by 1% agarose gels. Based on the concentration, DNA was diluted to 1 ng/µL with sterile water. V4 region of 16S rRNA genes was amplified with specific primer V4: 515F-806R. PCR mixture contained 10 ng target DNA, 15 µL of Phusion® High-Fidelity PCR Master Mix (New England Biolabs), and 0.2 µM of each primer, and cycling condition was set up including a first denaturation step for 1 min at 98 °C, followed by 30 cycles at 98 °C for 10 s, 50 °C for 30 s, and 72 °C for 30 s and a final extension for 5 min at 72 °C. PCR products were purified with Qiagen Gel Extraction Kit (Qiagen, Germany). Using NEBNext® Ultra™ IIDNA Library Prep Kit (Cat No. E7645) to generate the sequencing libraries and sequenced on the Illumina HiSeq 2500 platform. The generated 250 bp paired-end reads were truncated and then obtained high-quality clean tags via filtering and splicing. Compare clean tags with the database (Silva database https://www.arb-silva.de/) to obtain Amplicon Sequence Variants (ASVs). Subsequent analysis was all performed based on ASVs. Simpson, Pielou_e, Chao1, and Shannon indexes were performed to evaluate for alpha diversity. Principal coordinate analysis (PCoA) and Non-Metric Multi-Dimensional Scaling (NMDS) based on UniFrac distances were performed to assess microbial community difference among different groups. LEfSe analysis (LDA score threshold: 4) was to find out the biomarkers. Metastats analysis was to explore the differential bacteria between groups.

### Targeted SCFAs quantitative analysis

The level of SCFAs was measured with GC–MS analysis. Fecal samples (200 mg) were supplemented with 900 μL 0.5% phosphoric acid, vortexed and then centrifuged at 14,000*g* for 10 min. Then 800 μL of the supernatant was mixed with an equal volume of ethyl acetate, vortexed (2 min) and centrifuged (14,000×*g*, 10 min). 500 μL of the supernatant was supplemented with 500 μM of 4-methylpentanoic acid as internal standards and then extracted according to the manufacturer's protocol (Novogene Biotech Co., Ltd., Beijing, China). The extracted samples were separated by Agilent DB-WAX capillary column (30 m × 0.25 mm ID × 0.25 µm) gas chromatography system and detected using the 7890A/5975C GC–MS system (Agilent, USA). MSD Chemstation software was used to extract the chromatographic peak area and retention time. The concentrations of SCFAs were acquired according to the standard curves.

### Cytokines microarray assay

The colon tissues of 1 cm length collected from the same distal colon area were used for inflammatory factors gene expression profiles analysis using mouse Cytokines &Chemokines qPCR Array according to the manufacturer’s protocol (Wcgene Biotech, Shanghai, China). Data were analyzed using Wcgene Biotech software. Genes with fold-changes more than 1.3 and *P* < 0.05 were considered to be of biological significance.

### Enzyme-linked immunosorbent assay (ELISA)

Approximately 100 mg colon tissues were weighed and put into 1 mL cold PBS followed by trituration and centrifugation at 5000*g* for 10 min to obtain colon tissue homogenate. Cytokines including IL-10 (EK210/4-96; MULTI SCIENCES), CCL5/RANTES (EK2129/2-96; MULTI SCIENCES), IL-17A (EK217/2-96; MULTI SCIENCES), IL-1β (EK201B/3-96; MULTI SCIENCES), and IL-6 (EK206/3-96; MULTI SCIENCES) in the colon tissue homogenate were measured by ELISA Kits according to the manufacturer’s instructions. The concentrations of cytokines were obtained according to the standard curves. Results were expressed as pg/mL of colon homogenates in each sample.

### Mononuclear cell isolation

Cells from colonic lamina propria (LP), mesenteric lymph nodes (MLN) and spleen were isolated as described previously [22, 27]. Briefly, MLN and spleen were mechanically dissociated in ice-cold PBS. The resulting cell suspensions were passed through a 70-μm mesh cell strainer, and spleen also needed erythrocyte lysis. To prepare colonic LP cells, associated fat and Peyer’s patches were removed, the colonic tissue was washed in cold PBS to remove fecal contents and cut open longitudinally, and the colon was cut into 2-cm pieces and washed twice in 2 mL PBS containing 1 mM dithiothreitol, 10% FCS and 1 mM EDTA with constant agitation for 40 min at 37 °C. And then the pieces were minced exactly 50 times to obtain 1 mm tissue fragments and incubated in 2 mL of RPMI-1640 supplemented with 2.5 mg/mL Hyaluronidase (Biosharp), 1.5 mg/mL Collagenase II (Biosharp), and 0.25 mg/mL DNase I (Solarbio) with constant agitation for 45 min at 37 °C. The cells suspension was extracted through filtration and centrifugation, and the resuspended pellet was further purified from the interface of a 45%/72% Percoll density gradient.

### Flow cytometry analysis

Single-cell preparations were stained for Treg cells with surface antibodies to the following markers: APC/Cy7-anti-mouse CD4 (100413; Biolegend), PE/Cy7-anti-mouse CD25 (102015; Biolegend). While the single-cell suspensions used to stain Th cells needed to be incubated with Cell Activation Cocktail (Biolegend, San Diego, USA) in 5% CO2 at 37 °C for 6 h. After stimulation, the cells were stained with anti-CD4. For intracellular staining, all cells were fixed for 40 min in BD Fix/Perm buffer and washed in BD Perm/Wash buffer twice, and then incubated with FITC-anti-mouse IFN-γ (AM0IF01-20; MULTI SCIENCES), PE-anti-mouse IL-17A (AMO1704-20; MULTI SCIENCES), APC-anti-mouse IL-4 (AMOI405-20; MULTI SCIENCES), or PE-anti-mouse FOXP3 (126403; Biolegend) for 30 min at 4 °C. Stained cells were tested on a FACSVerse flow cytometer (BD Biosciences, San Jose, CA) and analyzed using FlowJo software (TreeStar, USA).

### Statistical analysis

Data were analyzed using GraphPad Prism version 8.0 (GraphPad Software, San Diego, CA, USA) and results were shown as mean ± SD. Differences between the mean values for groups were analyzed by an unpaired two-tailed Student’s *t*-test. Comparisons of parameters for three groups were made by one-way analysis of variance (ANOVA) followed by Tukey's test. Pearson statistical method was applied for the correlation analysis. A value of *P* < 0.05 was considered statistically significant.

## Results

### Phenotype identification of HUMSCs

**Fig. 1:**
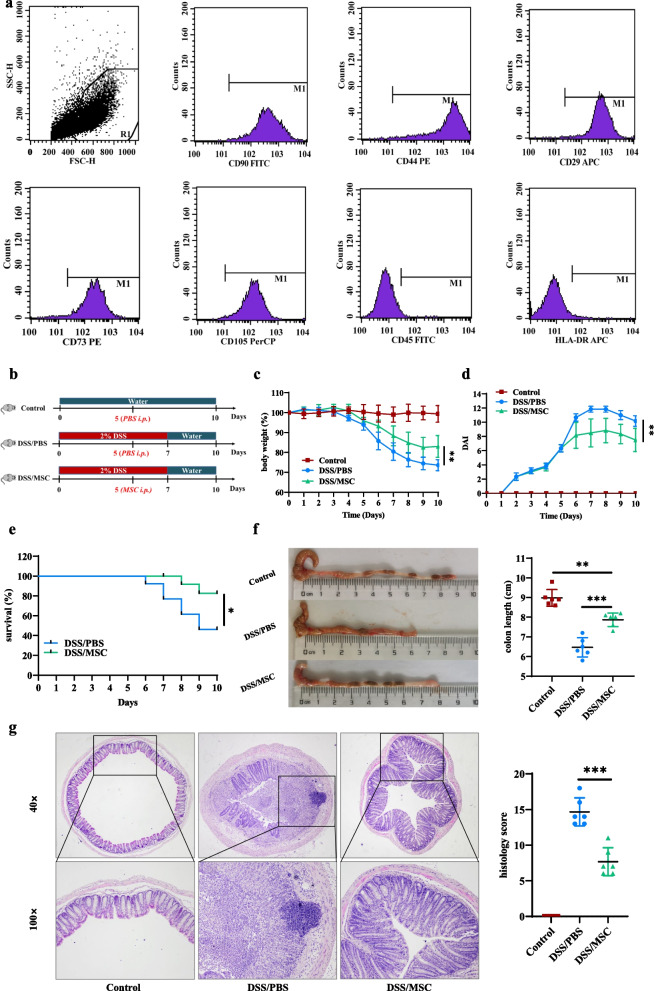
HUMSCs ameliorated DSS-induced experimental colitis. **a** The phenotype of MSCs was identified with high expression of MSCs markers, including CD90, CD44, CD29, and CD73, CD105, but negative for the expression of leukocyte markers, including CD45 and HLA-DR. **b** Diagram illustrating the experimental design employed in this study. Mice were treated with 2% DSS in their drinking water for 7 days followed by 3 days of normal water. MSCs (1 × 10^6^ cells) suspended in PBS were administered by intraperitoneal (i.p.) injection in DSS/MSC group mice on day 5 of the study. While the Control and DSS groups of mice were administered with the same volume of PBS by i.p. injection. **c** Daily body weight change (n = 6). **d** Disease activity index (DAI) score (n = 6). **e** Survival (n = 13). **f** Representative pictures of colon and colon length (n = 6). **g** Representative microscopic pictures of H&E staining (40× and 100× magnification) and histopathological score (n = 6). Data were presented as means ± SD. *P* values were calculated by one-way analysis of variance (ANOVA) followed by Tukey's test, *p < 0.05; **p < 0.01; ***p < 0.001

As shown in Fig. 1a, the cells harvested at the third passage were high expression of MSCs markers, including CD90, CD44, CD29, CD73, and CD105 but negative for the expression of leukocyte markers, including HLA-DR and CD45. The results showed that the cells were HUMSCs and could be used for subsequent experiments.

### HUMSCs administration ameliorated DSS-induced colitis

Murine DSS-induced colitis could efficiently simulate the typical clinical phenotypes of IBD [28]. To investigate the therapeutic effect of HUMSCs on DSS-induced colitis, experimental colitis was induced in mice by administering 2% DSS in water continuously for 7 days followed by 3 days of normal water. While HUMSCs suspended in PBS were administered by intraperitoneal injection in DSS/MSC group on day 5 (Fig. 1b). Compared with the DSS/PBS group, HUMSCs administration prominently ameliorated DSS-induced colitis, as evidenced by the distinct recovery of weight loss (Fig. 1c), decreased DAI score (Fig. 1d), increased survival rate (Fig. 1e), and relieved colon shortening (Fig. 1f). H&E staining was performed to systematically evaluate the severity of colonic mucosa injury. Compared with DSS/PBS group mice showing more loss of crypts, infiltration of mononuclear cells, severer damage of goblet cells, and higher histopathological score, DSS/MSC group mice exhibited relatively intact colonic architecture, mild mucosal damage, less mononuclear cells infiltration, and lower histopathological score. (Fig. 1g).

### HUMSCs shaped intestinal immune responses through regulating Treg/Th2/Th17 balance and altering the cytokine profile

**Fig. 2:**
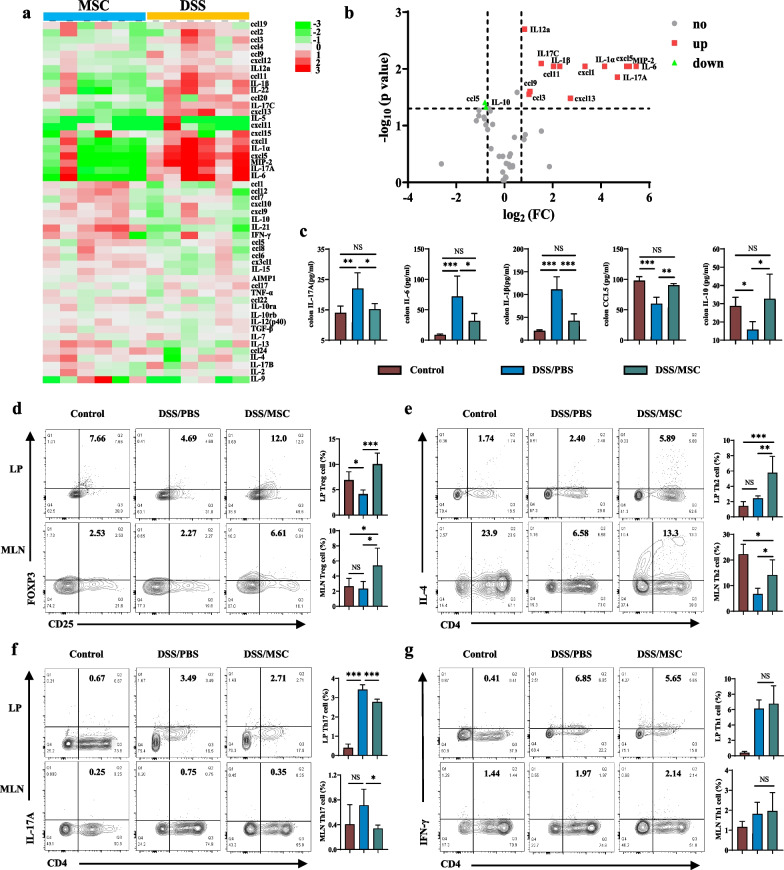
HUMSCs shaped intestinal immune responses through regulating Treg/Th2/Th17 balance and altering the cytokine profile. **a** Heatmap of cytokine mRNA relative expression levels from colon tissue measured by cytokine microarray. **b** Volcano plot of cytokine differential expression from colon tissue in DSS/PBS group compared to DSS/MSC group (fold-change > 1.3, *P* < 0.05). n = 6 each group. *p* values were calculated using Unpaired T-test. **c** IL-17A, IL-6, IL-1β, CCL5, and IL-10 cytokines levels in colon tissue homogenate were measured by ELISA among Control, DSS/PBS and DSS/MSC groups (n = 6). Treg (CD4^+^CD25^+^Foxp3^+^) (**d**), Th2 (CD4^+^IL-4^+^) (**e**), Th17 (CD4^+^IL-17A^+^) (**f**), and Th1 (CD4^+^IFN-γ^+^) (**g**) cells in the colonic LP and MLN from Control, DSS/PBS and DSS/MSC groups were analyzed by flow cytometry and bar charts of the percentages of cells were displayed (n = 5). Data were presented as means ± SD. *P* values were calculated by ANOVA followed by Tukey's test. *p < 0.05; **p < 0.01, ***p < 0.001, NS indicates *p* > 0.05

Considering the fact that cytokines, including pro- and anti-inflammatory factors, are key effector molecules for changes in intestinal immune homeostasis [5], cytokine microarray was conducted to detect the cytokines profile changes in intestinal mucosa following HUMSCs administration. The alterations of 50 cytokines were shown by the heatmap (Fig. 2a), and the differentially expressed cytokines were displayed by the volcano plot (Fig. 2b). Compared with DSS group, the pro-inflammatory cytokines manifested significantly decreasing trend in HUMSCs group, especially IL-17A, IL-17C, IL-6, IL-1α, IL-1β, IL-12a, MIP-2, cxcl5, cxcl1, cxcl13, ccl11, ccl9, ccl3. Instead, the anti-inflammatory cytokines, including CCL5 and IL-10, were significantly upregulated in HUMSCs group (Fig. 2a, b). The differentially expressed cytokines were furtherly validated by ELISA at the protein level. Among them, IL-17A, IL-6, IL-1β, CCL5, and IL-10 manifested significant differences between the two groups (Fig. 2c), which were consistent with the cytokine microarray results.

Given the markedly altered cytokines profile after HUMSCs treatment, and CD4^+^ T cells associated with cytokine secretion especially Th1, Th2, Th17 and Treg cells as the principal effector cells involved in the development of IBD [5], T cell responses were phenotyped by flow cytometry. In LP and MLN, the percentages of Treg (CD4^+^CD25^+^Foxp3^+^) (Fig. 2d) and Th2 (CD4^+^IL4^+^) (Fig. 2e) cells were significantly enhanced with HUMSCs treatment, and Th17 cells (CD4^+^IL-17A^+^) (Fig. 2f) were suppressed in HUMSCs-mice, and while the effect on Th1 (CD4^+^IFN-γ^+^) cells differentiation was not observed with HUMSCs application (Fig. 2g). Consistently, DSS/MSC group had the similar tendencies in the absolute numbers of Treg, Th2, Th17, and Th1 cells compared with DSS/PBS group mice in LP and MLN (Additional file 1: Fig. S1). However, the T cells responses showed no difference following HUMSCs administration in spleen (Additional file 1: Fig. S2).

### HUMSCs treatment regulated the composition of gut microbiota

**Fig. 3:**
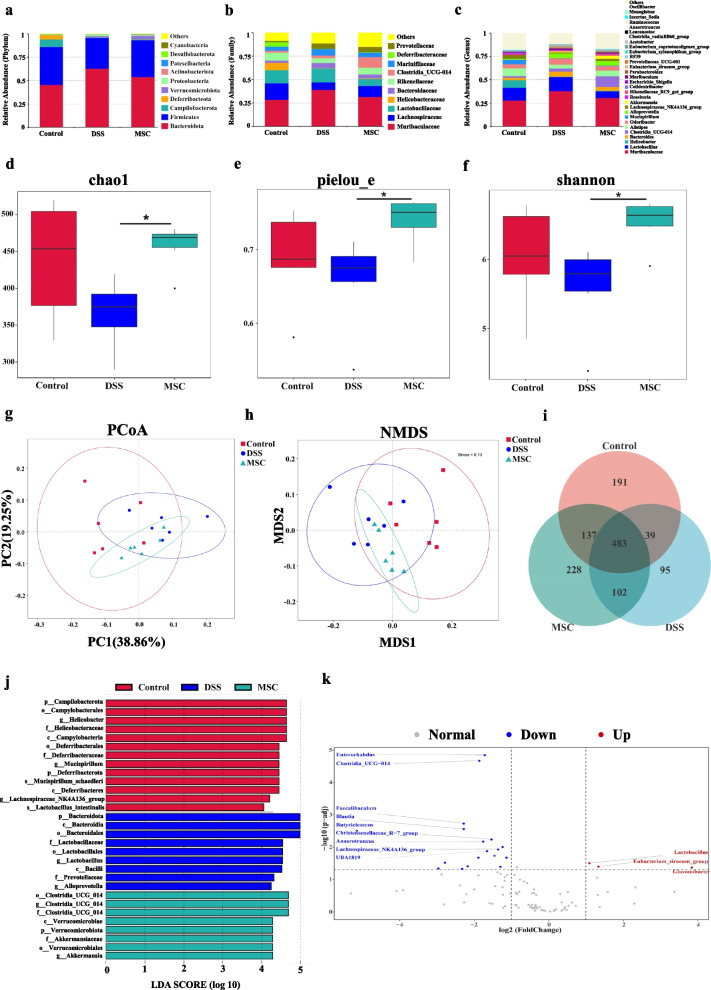
HUMSCs treatment significantly remodeled the gut microbiota diversity and composition. Histograms of relative abundance for top 10 microbes at the phylum (**a**) and family (**b**) levels. **c** Histograms of relative abundance for top 30 microbes at the genus level. Alpha diversity boxplot of chao1 index (**d**), pielou_e index (**e**), and shannon index (**f**). **g** Principal coordinate analysis (PCoA) based on weighted UniFrac distances for beta diversity. **h** Non-Metric Multi-Dimensional Scaling (NMDS) based on weighted UniFrac distances for beta diversity. **i** Venn diagram displaying the common and unique ASVs among Control, DSS/PBS and DSS/MSC groups. **j** Analysis of differences in the microbial taxa among the three groups was shown using LEfSe (LDA coupled with effect size measurements). **k** Based on the ASVs abundance at the genus level, a volcano plot was organized for the analysis of differential microbiota between DSS/PBS and DSS/MSC groups using Metastats analysis (fold-change > 2, *P* < 0.05). Blue nodes denote significantly up-regulated genera in DSS/MSC group, and red nodes represent significantly up-regulated genera in DSS/PBS group. n = 6 mice per group

Next, we further explored the impact of HUMSCs on the gut microbial composition of DSS-treated mice via 16S rRNA sequencing. We first assessed the difference of populations and abundances among the three groups. The top 10 microbes at the phylum (Fig. 3a), class (Additional file 1: Fig. S3a), order (Additional file 1: Fig. S3b), family (Fig. 3b) and the top 30 microbes at genus (Fig. 3c) levels were shown and indicated significant variations in the landscape of gut microbiota. At the phylum level (Fig. 3a), *Bacteroidota* and *Firmicutes* were predominant phyla in the fecal microbiota. Compared with Control mice, DSS administration increased *Bacteroidota*, *Proteobacteria* and decreased *Firmicutes*, and HUMSCs treatment reversed this trend, decreasing *Bacteroidota*, *Proteobacteria* and increasing *Firmicutes* (Additional file 1: Fig. S3c). At the family level (Fig. 3b), the fecal microbiota was dominated by *Muribaculaceae, Lachnospiraceae, Lactobacillaceae and Bacteroidaceae*, and we observed an obvious increase in *Muribaculaceae*, *Bacteroidaceae* and decrease in *Lachnospiraceae* in DSS group, which were restored with HUMSCs treatment similar to control group (Additional file 1: Fig. S3d). At the genus level (Fig. 3c), HUMSCs also restored the change in *Muribaculaceae* bacteria community induced by DSS drinking. Notably, we still observed a significant increase of abundance in *Akkermansia* and *Clostridia_UCG_014* with HUMSCs intervention than only DSS-treated mice or control mice (Additional file 1: Fig. S3e).

Subsequently, to evaluate the differences of community distribution within the groups, alpha diversity was calculated. Different indices including chao1 (Fig. 3d), observed_otus (Additional file 1: Fig. S4a), pielou_e (Fig. 3e), shannon (Fig. 3f) and simpson (Additional file 1: Fig. S4b) manifested similar tendencies and found that HUMSCs-treated mice harbored a higher diversity by which to make fecal microbiota much closer to control. To assess the complexity of the community composition and compare the differences between groups, beta diversity was calculated based on UniFrac distance algorithm. Both models of weighted PCoA (Fig. 3g) and NMDS (Fig. 3h) were performed to visualize differences of samples in complex multi-dimensional data, which showed an obvious separation in the gut microbial structure between normal controls and mice with colitis (R = 0.524, *P* = 0.005), and a separation was also observed between DSS/PBS group and DSS/MSC group (R = 0.167, *P* = 0.045). However, the community composition was relatively similar between control mice and MSC-treated mice (R = 0.207, *P* > 0.05). In addition, according to the ASVs results obtained from denoise, the common and unique ASVs were analyzed among groups. As shown in Fig. 3i, the overlapping number of ASVs between the MSC and control groups was 3.5 times higher than that between the colitis and control groups (137 vs 39), suggesting that HUMSCs largely restored the dysregulated microbiota in colitis mice with similarity to normal mice.

To confirm which bacterium was altered by HUMSCs treatment and in turn affected the disease progression against DSS-induced colitis, linear discriminant analysis (LDA) of the effect size (LEfSe) was conducted to identify differentially abundant fecal bacterial taxa. As shown in Fig. 3j, the genus *Lactobacillus* and the genus *alloprevotella* were the dominant differential bacteria resulting in gut microbiota dysbiosis in the DSS group, and while these types of taxa were down-regulated with HUMSCs administration (Additional file 1: Fig. S5a). More notably, several bacterial genera including *Akkermansia (family Akkermansiaceae; order Verrucomicrobiales; class Verrucomicrobiae; phylum Verrucomicrobia)* and *Clostridia_UCG_014 (family and order Clostridia_UCG_014)* were enriched after HUMSCs treatment (Fig. 3j; Additional file 1: Fig. S5b). Additionally, based on the ASVs abundance at the genus level, a volcano plot was also organized for the analysis of differential microbiota between DSS/PBS and DSS/MSC groups (Fig. 3k). Similarly, the *Lactobacillus* displayed a relatively high abundance in the DSS/PBS group, while the *Clostridia_UCG_014* was significantly enriched in the DSS/MSC group, which was consistent with the LEfSe analysis results. Meanwhile, the abundance of *Faecalibaculum*, *Blautia*, *Anaerotruncus*, and *Lachnospiraceae_NK4A136_group* also displayed significant dominance with HUMSCs administration. Collectively, HUMSCs treatment significantly remodeled the gut microbiota diversity and composition, reversing DSS-induced intestinal dysbiosis.

### The protective effect of HUMSCs on colon inflammation was abrogated by antibiotic treatment

**Fig. 4:**
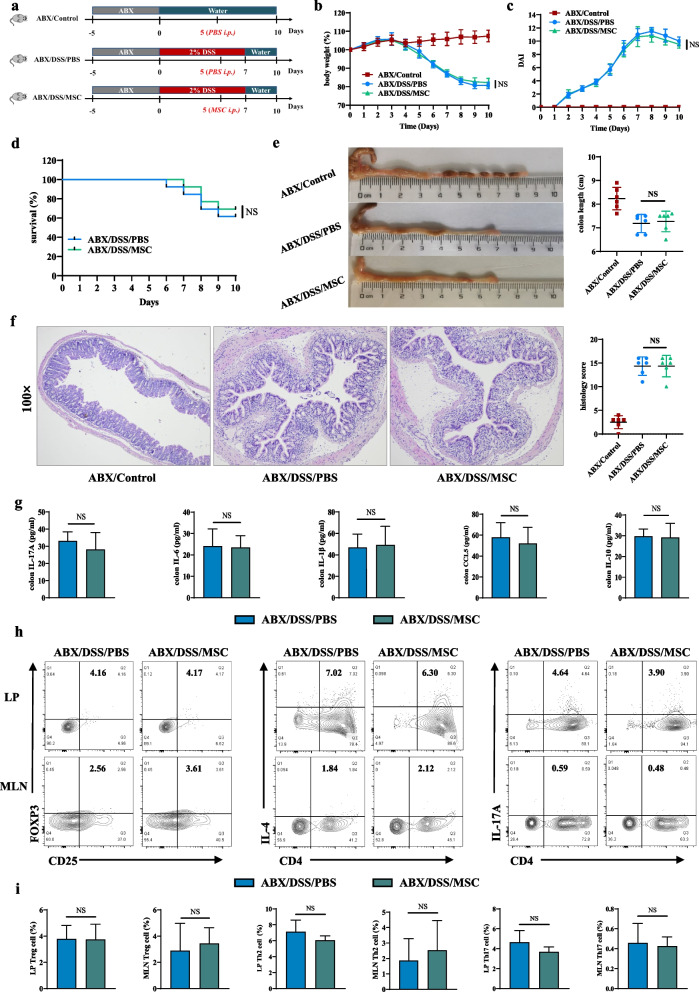
The protective effect of HUMSCs on colon inflammation was abrogated by antibiotic treatment. **a** Diagram illustrating the mouse model of colitis employed in this study. Wild-type mice were gavaged using broad-spectrum antibiotic (ABX) once a day for 5 days to deplete gut microbiota, and then 2% DSS drinking water was administered for 7 days to induce colitis. ABX/DSS/MSC group mice were treated with 1 × 10^6^ HUMSCs (i.p.) on day 5, and ABX/DSS/PBS and ABX/Control group mice were delivered an equal volume of vehicle in the same way. **b** Daily body weight change (n = 6). **c** DAI score (n = 6). **d** Survival (n = 13). **e** Representative pictures of colon and colon length (n = 6). **f** Representative microscopic pictures of H&E staining (100× magnification) and histopathological score (n = 6). **g** IL-17A, IL-6, IL-1β, CCL5, and IL-10 cytokines levels in colon tissue homogenate were measured by ELISA between ABX/DSS/PBS and ABX/DSS/MSC groups (n = 6). **h** Representative flow cytometric analysis of Treg, Th2, and Th17 cells in the colonic LP and MLN from ABX/DSS/PBS and ABX/DSS/MSC groups. **i** Bar charts of the percentages of Treg, Th2, and Th17 cells in the colonic LP and MLN were displayed (n = 5). Data were presented as means ± SD. *P* values were calculated using Unpaired T-test, NS indicates *p* > 0.05

To investigate whether gut microbiota participated in the protective effect of HUMSCs against colon inflammation, wild-type mice were gavaged using broad-spectrum antibiotic (ABX) for gut microbiota depletion followed by DSS treatment (Fig. 4a). Strikingly, ABX/DSS/PBS group mice displayed similar severity of colitis to ABX/DSS/MSC group, as evidenced by indistinguishable weight change (Fig. 4b), DAI score (Fig. 4c), survival rate (Fig. 4d), colon shortening (Fig. 4e), and histopathology score (Fig. 4f) following gut flora depletion. Furthermore, the inflammatory responses in the ABX/DSS/MSC group showed comparable tendencies to those in the ABX/DSS/PBS group, as evidenced by similar levels of proinflammatory cytokines including IL-17A, IL-6, IL-1β, and anti-inflammatory cytokines CCL5, IL-10 (Fig. 4g). Meanwhile, to further explore the improved effect on Treg/Th2/Th17 balance with HUMSCs application in gut microbiota-depleted mice, mononuclear cells isolated from colonic LP and MLN were phenotyped by flow cytometry. The frequencies of Treg, Th2, and Th17 cells displayed no significant difference between the ABX/DSS/PBS and ABX/DSS/MSC groups (Fig. 4h–i). Consistently, the absolute numbers of these CD4^+^ T lymphocyte subsets also revealed corresponding tendencies after gut microbiota depletion (Additional file 1: Fig. S6), indicating that the presence of gut microbiota was indispensable for the protective effects of HUMSCs on the intestinal immune homeostasis in colitis.

### Fecal microbial transplants from HUMSCs-treated mice inhibited colitis

**Fig. 5:**
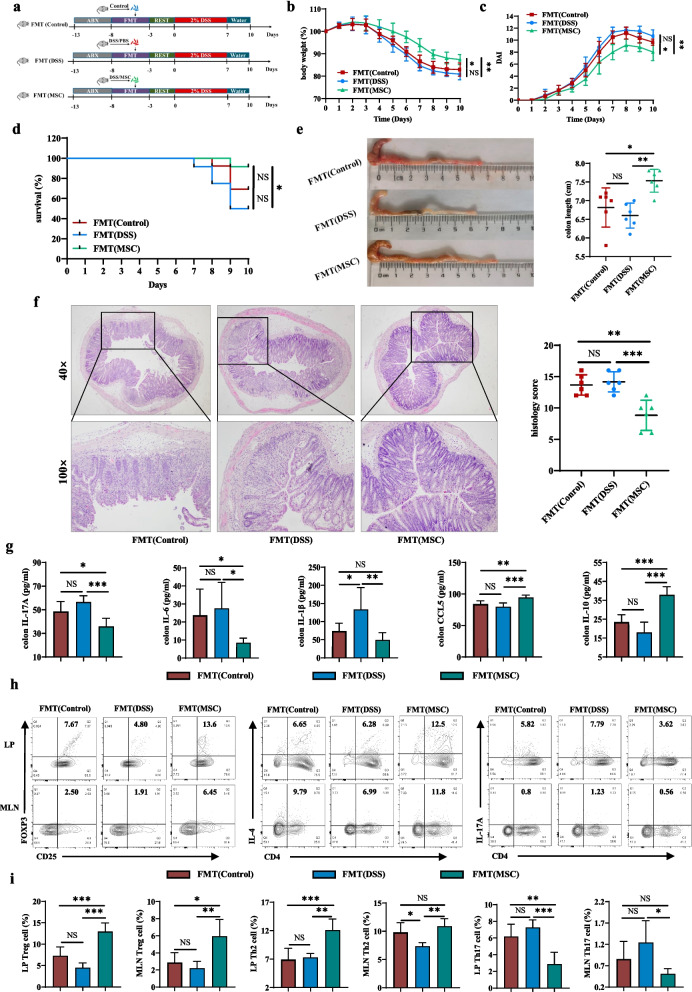
Fecal microbial transplants from HUMSCs-treated mice inhibited colitis. **a** Diagram illustrating the mouse model of colitis employed in this study. The feces from Control, DSS/PBS and DSS/MSC groups mice were transplanted into ABX-mice via intragastric administration once a day for 5 days, and then followed by DSS treatment after three days of rest. **b** Body weight change (n = 6). **c** DAI score (n = 6). **d** Survival (n = 12). **e** Representative pictures of colon and colon length (n = 6). **f** Representative microscopic pictures of H&E staining (40× and 100× magnification) and histopathological score (n = 6). **g** IL-17A, IL-6, IL-1β, CCL5, and IL-10 cytokines levels in colon tissue homogenate were measured by ELISA among FMT(Control), FMT(DSS), and FMT(MSC) groups (n = 6). **h** Representative flow cytometric analysis of Treg, Th2, and Th17 cells in the colonic LP and MLN from FMT(Control), FMT(DSS), and FMT(MSC) groups. **i** Bar charts of the percentages of Treg, Th2, and Th17 cells in the colonic LP and MLN were displayed (n = 5). Data were presented as means ± SD. *P* values were calculated by ANOVA followed by Tukey's test, *p < 0.05; **p < 0.01; ***p < 0.001, NS indicates *p* > 0.05

To further confirm the causal role of the gut microbiota in the therapeutic effects of HUMSCs, we transplanted the feces of Control, DSS/PBS and DSS/MSC groups mice into ABX-mice via intragastric administration once a day for 5 days, and then followed by DSS treatment after three days of rest (Fig. 5a). FMT(MSC) recipient mice experienced significantly reduced colitis, manifested as reduced weight loss (Fig. 5b), decreased DAI score (Fig. 5c), higher survival rate (Fig. 5d), less colon shortening (Fig. 5e), as well as significantly decreased colon inflammation and lower histopathology score (Fig. 5f) than FMT (DSS) recipient mice. Additionally, colon tissues from FMT(MSC) group mice showed less protein levels of IL-17A, IL-6, and IL-1β, and higher IL-10, CCL5 in accordance with that of MSC itself (Fig. 5g). As expected, the balance of Treg/Th2/Th17 in colitis mice was also protected by FMT from MSCs-treated mice, which was confirmed by increasing the percentages of Treg and Th2 cells, while decreasing the ratio of Th17 cells both in LP and MLN (Fig. 5h–i), and the consistent tendencies were also observed in the absolute numbers (Additional file 1: Fig. S7). Taken together, HUMSCs ameliorated DSS-induced colitis in a gut microbiota-dependent manner, wherein the changed gut microbiota following HUMSCs treatment was responsible for the maintenance of intestinal immune homeostasis.

### HUMSCs administration transformed microbiota-derived short-chain fatty acids (SCFAs) profiles

**Fig. 6:**
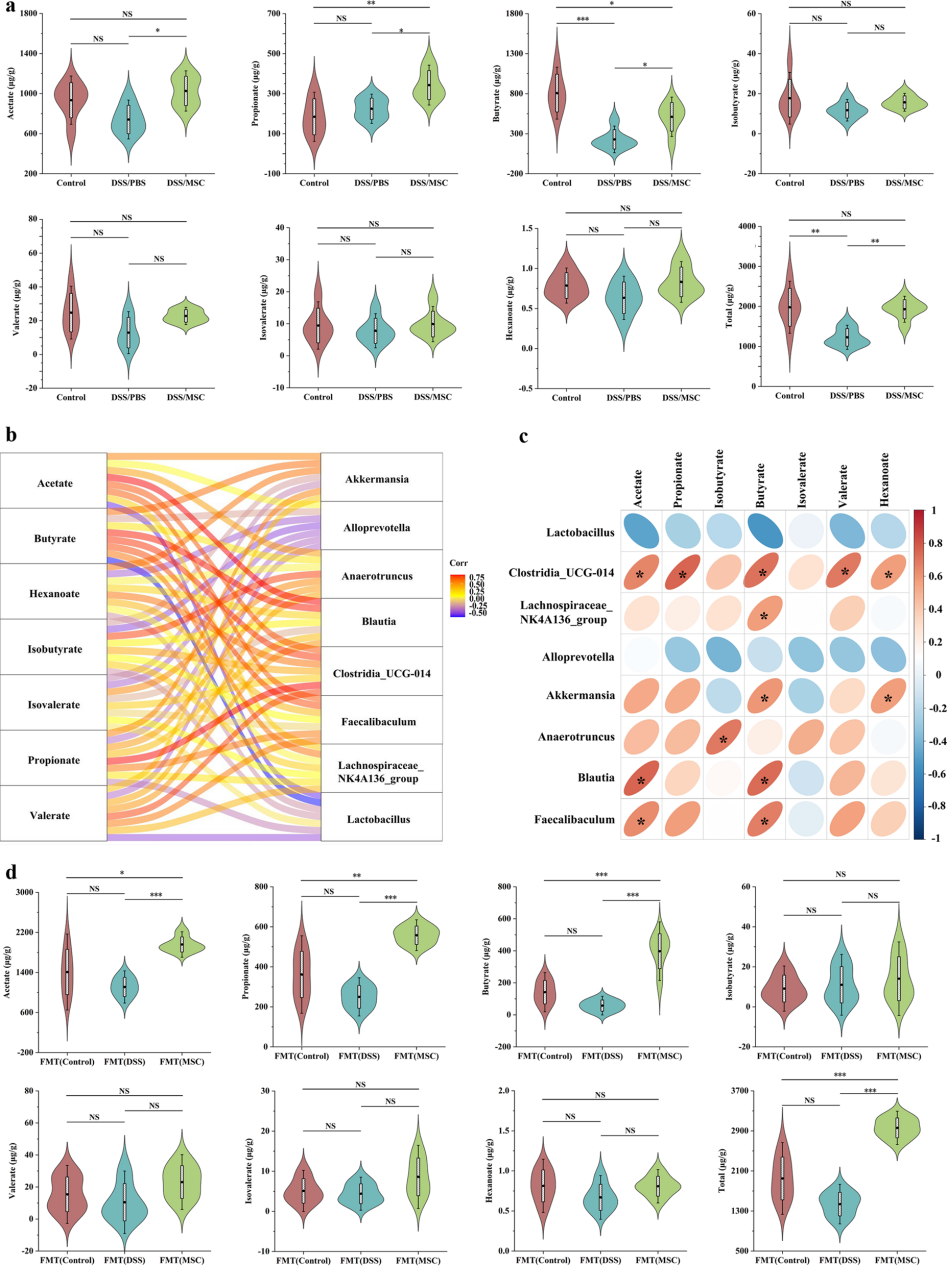
HUMSCs administration transformed microbiota-derived short-chain fatty acids (SCFAs) profiles. **a** Fecal SCFAs concentrations from the Control, DSS/PBS and DSS/MSC groups mice (n = 6). Data were presented as means ± SD. *P* values were calculated by ANOVA followed by Tukey's test, *p < 0.05; **p < 0.01; ***p < 0.001, NS indicates *p* > 0.05. **b** Sankey diagram displaying visually the association between gut microbes and fecal SCFAs profiles. The connection line represents the correlation, the red color denotes the positive correlation, and the blue denotes the negative correlation. **c** Heatmap showing the correlation between differentially enriched microbes and SCFAs profiles. The red color denotes a positive correlation, while blue color denotes a negative correlation. The intensity of the color is proportional to the strength of Pearson correlation. *P < 0.05. **d** Fecal SCFAs concentrations from the FMT (Control), FMT (DSS) and FMT (MSC) groups mice (n = 5). Data were presented as means ± SD. *P* values were calculated by ANOVA followed by Tukey's test, *p < 0.05; **p < 0.01; ***p < 0.001, NS indicates *p* > 0.05

Previous 16S rRNA sequencing analysis showed that gut microbiota in the DSS/MSC group displayed a predominance of the genera *Akkermansia, Clostridia_UCG_014, Faecalibaculum*, *Blautia*, *Anaerotruncus*, and *Lachnospiraceae_NK4A136_group*, which are associated with SCFAs metabolism [29–32]. SCFAs are the end products of gastrointestinal anaerobic fermentation and serve as major players in the maintenance of intestinal immune homeostasis [12]. Thus, to further explore whether HUMSCs treatment had an impact on bacterial metabolic output, the SCFAs profiles in fecal samples were evaluated by a targeted metabolomics assay. The chromatographic separation of metabolites was perfect, and the peak shape was sharp and symmetrical (Additional file 1: Fig. S8), which could be used for mass spectrometry quantification of each metabolite. As shown in Fig. 6a, Consistent with the changes in microbial community structure and compositions, HUMSCs administration markedly altered microbiota-derived SCFAs profiles, especially upregulating acetate, propionate and butyrate production, while the concentrations of isobutyrate, valerate, isovalerate and hexanoate displayed no significant difference between DSS/MSC and DSS/PBS groups. Meanwhile, the concentrations of total SCFAs in MSC-treated mice also manifested higher amounts. Pearson correlation analysis was further performed to understand the association between differentially enriched microbes and SCFAs profiles (Fig. 6b). Correlation analysis revealed that six differential bacterial genera enriched in MSC-treated group showed positive correlation with SCFAs. Especially the genera *Clostridia_UCG_014* had significant positive correlation with various SCFAs including acetate (r = 0.654, *P* = 0.021), propionate (r = 0.75, *P* = 0.005), butyrate (r = 0.714, *P* = 0.009), and valerate (r = 0.695, *P* = 0.012). *Akkermansia* displayed a strong positive correlation with butyrate (r = 0.596, *P* = 0.041) and hexanoate (r = 0.580, *P* = 0.048), and *Faecalibaculum* showed obvious positive correlation with acetate (r = 0.636, *P* = 0.026) and butyrate (r = 0.678, *P* = 0.015). However, the bacteria *Lactobacillus* and *alloprevotella* enriched in the colitis group manifested negative correlation with SCFAs, although the statistical differences were not significant (*p* > 0.05) (Fig. 6c).

To furtherly validate whether the increased metabolite SCFAs production originated from gut microbiota changes following HUMSCs administration, targeted metabolomics assay was also conducted among FMT groups. As expected, the FMT(MSC) recipient mice showed the marked upregulation of acetate, propionate, and butyrate, with the consistent trends to that of DSS/PBS and DSS/MSC groups (Fig. 6d).

Taken together, alteration of the gut microbiota and increased production of SCFAs in mice with colitis occurred in response to HUMSCs administration, suggesting that gut microbiota-derived metabolites SCFAs might play a critical role in alleviating DSS-induced colitis.

### Sterile fecal filtrate from HUMSCs-treated mice attenuated DSS-induced colitis

**Fig. 7:**
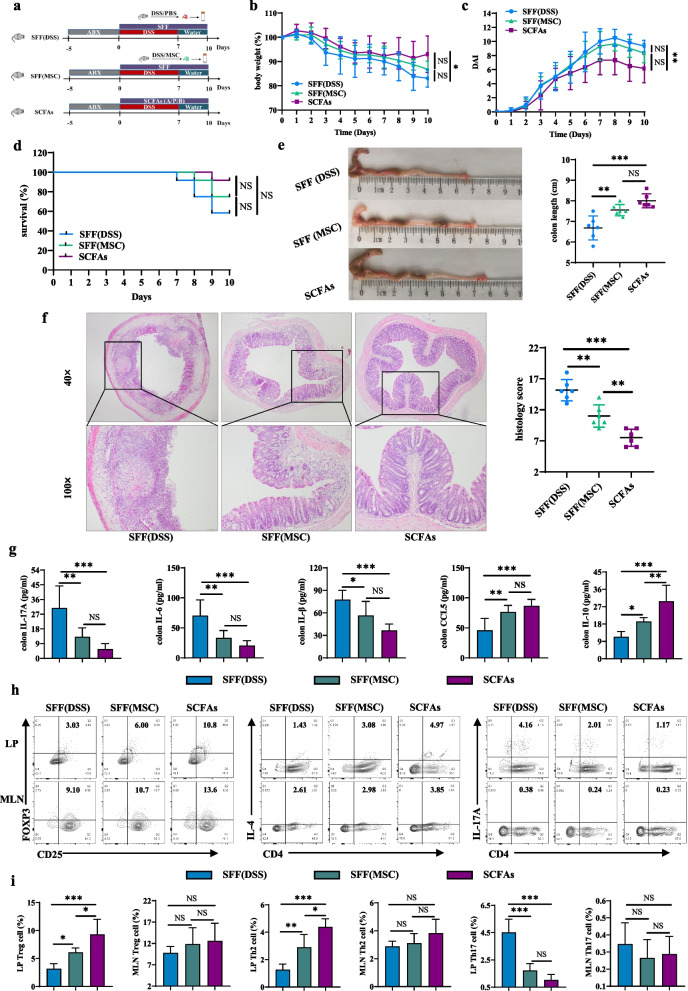
Sterile fecal filtrate (SFF) from HUMSCs-treated mice attenuated DSS-induced colitis. **a** Diagram illustrating the mouse model of colitis employed in this study. The fecal supernatant from DSS/PBS and DSS/MSC groups mice was centrifuged and then passed through 0.22 μm filters to obtain sterile fecal filtrate (SFF) with different concentrations of SCFAs. ABX-mice were then gavaged using SFF for continuous 10 days. And another group mice were orally administered with a mixture of SCFAs including acetate (A), propionate (P) and butyrate (B) as a positive control. **b** Body weight change (n = 6). **c** DAI score (n = 6). **d** Survival (n = 12). **e** Representative pictures of colon and colon length (n = 6). **f** Representative microscopic pictures of H&E staining (40× and 100× magnification) and histopathological score (n = 6). **g** IL-17A, IL-6, IL-1β, CCL5, and IL-10 cytokines levels in colon tissue homogenate were measured by ELISA among SFF(DSS), SFF(MSC) and SCFAs groups (n = 6). **h** Representative flow cytometric analysis of Treg, Th2, and Th17 cells in the colonic LP and MLN from SFF(DSS), SFF(MSC) and SCFAs groups. **i** Bar charts of the percentages of Treg, Th2, and Th17 cells in the colonic LP and MLN were displayed (n = 5). Data were presented as means ± SD. *P* values were calculated by ANOVA followed by Tukey's test, *p < 0.05; **p < 0.01; ***p < 0.001, NS indicates *p* > 0.05

To furtherly investigate the role of changes in microbiota-derived metabolites SCFAs following HUMSCs treatment in attenuating DSS-induced colitis, the fecal supernatant from DSS/PBS and DSS/MSC groups mice was centrifuged and then passed through 0.22 μm filters to obtain sterile fecal filtrate (SFF) with different concentrations of SCFAs. ABX-mice were then gavaged using SFF for continuous 10 days. And another group mice were orally administered with a mixture of SCFAs including acetate (A), propionate (P) and butyrate (B) as a positive control (Fig. 7a). As expected, mice gavaged with SCFAs manifested the best therapeutic effect (Fig. 7b–f). As exhibited by the less body weight loss, lower DAI score, less mortality, less shortening of colon, less colonic inflammation and histological score. Most notably, oral delivery of SFF from MSC-treated mice significantly reduced the severity of disease, as gaged by colon length and histopathology score, but not body weight, DAI score, or mortality, compared with SFF(DSS) group mice. Additionally, colonic tissue of mice that received SCFAs or the SFF from MSC-treated mice had significantly decreased IL-17A, IL-6, IL-1β and increased IL-10, CCL5 compared to those receiving SFF from DSS donor mice (Fig. 7g). Furthermore, the balance of Treg/Th2/Th17 in colitis mice was also restored via administration of SCFAs or SFF from MSC-treated mice, which was confirmed by increasing the percentages and the absolute numbers of Treg and Th2 cells, while decreasing Th17 cells in LP, but not in MLN (Fig. 7h–i; Additional file 1: Fig. S9).

**Fig. 8:**
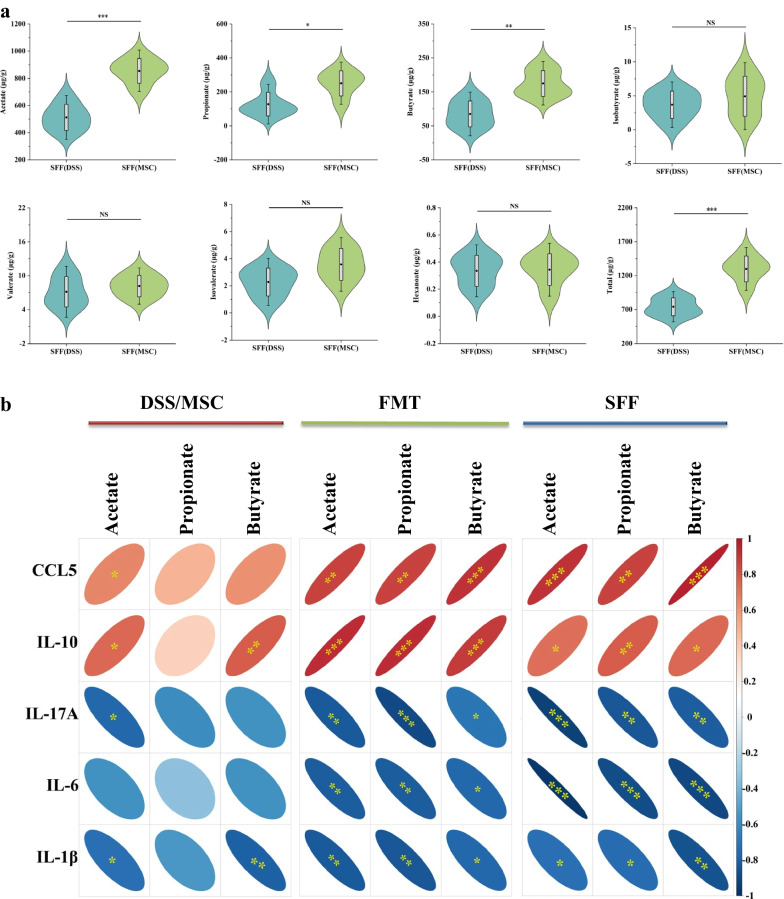
SCFAs regulated the immune inflammatory response in ABX-colitis mice. **a** Fecal SCFAs concentrations from the SFF(DSS) and SFF(MSC) groups mice (n = 5). Data were presented as means ± SD. *P* values were calculated using Unpaired T-test, *p < 0.05; **p < 0.01; ***p < 0.001, NS indicates *p* > 0.05. **b** Heatmap showing the correlation between SCFAs and cytokine among the three model systems including the model of colitis treated with HUMSCs or not, the FMT model, and SFF model systems. The red color denotes a positive correlation, while blue color denotes a negative correlation. The intensity of the color is proportional to the strength of Pearson correlation. *p < 0.05; **p < 0.01; ***p < 0.001

Meanwhile, compared with SFF(DSS) group mice, the SFF(MSC) group mice also displayed higher concentrations of acetate, propionate, and butyrate (Fig. 8a). To furtherly confirm the regulatory role of SCFAs on immune inflammatory response, the correlation between SCFAs and cytokine was analyzed. As the heatmap showed (Fig. 8b), in the model system of colitis treated with MSC or not, acetate level was significantly positively correlated with the expressions of CCL5 (r = 0.654, *p* = 0.040) and IL-10 (r = 0.741, *p* = 0.014), but inversely with IL-17A (r = -0.763, *p* = 0.010) and IL-1β (r = -0.729, *p* = 0.017). And butyrate was markedly positively correlated with IL-10 (r = 0.780, *p* = 0.008), but negatively with IL-1β (r = -0.797, *p* = 0.006). Noteworthily, in the FMT (Fig. 8b; Table 1) or SFF (Fig. 8b; Table 2) model system, the SCFAs, including acetate, propionate, and butyrate, all manifested prominently positive association with CCL5 (*p* < 0.05) and IL-10 (*p* < 0.05), and inversely with IL-17A (*p* < 0.05), IL-6 (*p* < 0.05), and IL-1β (*p* < 0.05). Which indicated that SCFAs played an important role in regulating immune inflammation as previously reported [12].

**Table 1:** The correlation between SCFAs and cytokine in FMT model system

Cytokine	SCFAs					
	Acetate		Propionate		Butyrate	
	r	*p*	r	*p*	r	*p*
CCL5	0.865	0.001	0.861	0.001	0.914	< 0.001
IL-10	0.930	< 0.001	0.932	< 0.001	0.882	< 0.001
IL-17A	− 0.821	0.004	− 0.883	< 0.001	− 0.699	0.025
IL-6	− 0.819	0.004	− 0.817	0.004	− 0.760	0.011
IL-1β	− 0.824	0.003	− 0.830	0.003	− 0.761	0.011

*SCFA* short-chain fatty acid, *FMT* fecal microbiota transplantation, *CCL5* C–C chemokine ligand 5, *IL* interleukin

**Table 2:** The correlation between SCFAs and cytokine in SFF model system

Cytokine	SCFAs					
	Acetate		Propionate		Butyrate	
	r	*p*	r	*p*	r	*p*
CCL5	0.919	< 0.001	0.860	0.001	0.967	< 0.001
IL-10	0.728	0.017	0.780	0.008	0.749	0.012
IL-17A	− 0.929	< 0.001	− 0.839	0.002	− 0.808	0.005
IL-6	− 0.965	< 0.001	− 0.873	< 0.001	− 0.890	< 0.001
IL-1β	− 0.723	0.018	− 0.731	0.016	− 0.853	0.002

*SCFA* short-chain fatty acid, *SFF* sterile fecal filtrate, *CCL5* C–C chemokine ligand 5, *IL* interleukin

Collectively, the benefit was pronounced with the SFF from MSC-treated mice. Thus, it was also reasonable to infer that HUMSCs ameliorated colon immune inflammation in a gut microbiota-dependent manner, wherein microbiota-derived SCFAs played a critical regulatory mechanism.

## Discussion

Besides host genetics, environmental factors especially gut microbiota, have been strongly associated with IBD initiation and progression [33]. Cooperation between host immunity and the gut bacteria is essential for maintaining intestinal homeostasis, where the metabolites derived from microbiota such as SCFAs may exert anti-inflammatory function [10]. MSCs therapy, as a novel strategy, has shown promising efficacy and safety in IBD field mainly via its powerful immunoregulation and the ability of tissue regeneration [13]. In addition, emerging evidence underlines a novel role of MSCs in the regulation of intestinal flora [16, 17], and we recently discovered that HUMSCs remodeled diversification of gut microbiota might via promoting the transformation of IgA^+^ plasmacytes in Peyer’s patches and the secretion of lgA in intestinal lumen [22]. Although the mechanism by which MSCs regulate the gut microbiota still remains to be elucidated, whether the protective effect of MSCs against colitis is related to gut microbiota has attracted the attention and deserves to be explored. The novel data herein revealed that the interaction existed among MSCs, host, and gut bacteria in the suppression of colitis, namely, MSCs alleviated colon inflammation in a gut microbiota-dependent manner and meanwhile in untangling this tripartite mechanism of inhibiting colitis we noted that microbiota-derived metabolites SCFAs displayed the critical role in mediating MSCs restoring CD4^+^T cell immune homeostasis.

MSCs from various sources, such as adipose, bone marrow, umbilical cord, etc., have shown outstanding effects in the treatment of IBD [34]. MSCs from umbilical cord were selected in the present study as we previously reported [20]. Historically, intravenous injection is the most common intervention for MSCs delivery, and while growing evidence suggests that the intraperitoneal injection displays better colitis restoration [35], more MSCs homing to inflamed colon but fewer trapped cells in lung, compared with intravenous injection [21]. Our previous study also showed a promising effect of intraperitoneal administration of HUMSCs on experimental colitis [22]. Thus, the intraperitoneal injection method continued to be employed in this study. In agreement with previous studies [22, 36], we confirmed that the therapeutic applications of intraperitoneal HUMSCs led to the alleviation in DSS-induced colitis, as indicated by the decreased weight loss and DAI score, and the increased survival and colonic length, as well the promoted recovery of histological damage.

Numerous studies have fully confirmed the importance of abnormal immune regulation in the occurrence and development of IBD, especially the adaptive immune response dominated by CD4^+^ T cells [6]. Expressing the specific transcription factor Foxp3, Treg cells perform immunosuppressive function to inhibit the activity of pro-inflammatory cells Th1 and Th17 by secreting IL-10, and Th2 cells can also show a synergistic effect with Treg cells [5, 6]. As previously evidenced [20], HUMSCs delivery markedly altered the cytokines profile through downregulating the level of pro-inflammatory cytokines as well as upregulating the immunosuppressive cytokines in DSS-induced colitis, especially for the pro-inflammatory cytokine of IL-17A, IL-6, IL-1β and anti-inflammatory cytokine of IL-10, CCL5. CCL5 (or RANKL) preferentially expressed by Treg cells, has been confirmed to exert immunosuppressive effects by inducing the generation of Treg cells [37]. And IL-6 and IL-1β, as typical pro-inflammatory cytokines, play a critical role in instructing the differentiation of Th17 cells [38]. Subsequently, considering the fact that the dynamic balance among CD4^+^T cell subsets plays an integral role in stabilizing gut homeostasis, T cell responses in the colonic LP, MLN were investigated by flow cytometry. We found that the Treg/Th2/Th17 balance was improved with HUMSCs administration, as exemplified by the increased frequencies of Treg and Th2 cells as well as decreased Th17 cells compared with the DSS group, and while Th1 cells response displayed no significant difference. Additionally, the absolute numbers of these T lymphocyte subsets also manifested the consistent tendencies. Which was in accordance with previous data [13], MSCs can regulate the immune response of CD4^+^ T cells and maintain intestinal immune tolerance, thereby suppressing colonic inflammatory responses.

Intestinal dysbiosis is considered the critical factor to break this homeostasis and promote the occurrence of IBD [4]. Enormous evidence has unveiled the presence of microbial imbalance in IBD patients, marked by the loss of symbiotic flora and the expansion of pathogenic bacteria [39]. Transfer fecal microbiota from IBD patients to germ-free mice induced colitis through perturbing the balance of Treg/Th2/Th17 [8], and while transplanting fecal flora from healthy people to mice with experimental colitis significantly improved the immune-inflammatory state of mice, reshaping Th1/Th17/Treg balance [9]. The maintenance of intestinal homeostasis depends on the coordinated interaction between the microbiota and the host immune system [40]. MSCs therapy for IBD primarily focus on host immunological processes, while has, until recently, overlooked the potential involvement of the gut microbiota as a regulator of mucosal immune homeostasis. Here, we observed that the microbiome disruption induced by DSS injury was to a great degree reversed by the administration of HUMSCs as previously reported [16, 22]. Originally, the abundance of microbes at different levels indicated significant variations in the landscape of gut microbiota, as characterized by the increased *Bacteroidota*, *Proteobacteria* and decreased *Firmicutes* in DSS-induced colitis mice, and while the imbalance of *Firmicutes/Bacteroidota* was restored by intraperitoneal HUMSCs more similar to normal mice. Our alpha diversity results based on the chao1, observed_otus, pielou_e, shannon and simpson indices manifested similar tendencies and found that HUMSCs-treated mice harbored a higher diversity by which to make fecal microbiota much closer to control. Beta diversity including PCoA and NMDS analysis indicated biological community structure alteration in mice treated with DSS drinking, and such the alteration was significantly remodeled more closely resembling that of healthy control group in mice treated with HUMSCs based on anosim statistical analysis. According to LEfSe analysis, the genus *Lactobacillus* and *Alloprevotella* were the dominant differential bacteria resulting in intestinal dysbiosis in the colitis mice consistently with previous research [41], and while these types of taxa were down-regulated with HUMSCs treatment. More interestingly, compared with control and DSS groups, HUMSCs application evidently promoted the intestinal enrichment of several bacterial genera including *Akkermansia*, *Clostridia_UCG_014*. *Akkermansia*, as a well-recognized next-generation probiotic and producer of SCFAs, has been proved to appear prominently in the correlations between gut microbes and host responses [42]. Its abundance is inversely correlated with IBD, metabolic disorders, and neurodegenerative disorders, but positively correlated with responses to immune checkpoint inhibitors in cancer immunotherapy [29, 43]. Multiple lines of evidences have shown that *Akkermansia* plays a crucial role in the progression of colitis, as proven by targeting host intestinal cells and contributing to the regulation of gut barrier function, antimicrobial peptide production, and the regulation of immune inflammation [29, 44]. Especially for CD4^+^ T cell immune homeostasis, *Akkermansia* favoures Treg cells expansion, which, in turn, controlled effector T cells to suppress disease symptoms [45]. *Clostridia_UCG_014*, also a probiotic producing SCFAs, displays the reduced abundance in IBD patients and animals [46, 47], which is negatively correlated with pro-inflammatory cytokines [48]. Meanwhile, the abundance of *Faecalibaculum*, *Blautia*, *Anaerotruncus*, and *Lachnospiraceae_NK4A136_group* also displayed significant dominance after HUMSCs treatment based on Metastats analysis, and all of them have been believed to be beneficial members that can regulate SCFAs metabolism and inversely associate with the occurrence of various diseases [30–32, 49, 50]. In particular, *Faecalibaculum*, also as a next-generation probiotic*,* with a lower concentration in IBD patients [51], maintains Th17/Treg balance and exerts significant anti-inflammatory effects in colitis rodents [52]. Accordingly, our results demonstrated that HUMSCs employment could modulate gut microbiota dysbiosis caused by DSS-induced colitis via increasing microbial biological diversity and promoting the relative abundance of beneficial bacteria. And it is speculated that HUMSCs may affect colitis immune repair through microbial modulation.

To explore whether the altered gut flora participates in the protective effect of MSCs on colitis, we depleted gut flora with antibiotic cocktail. Strikingly, the protective effect of HUMSCs on colitis disappeared following gut microbiota depletion, and the severity of colon inflammation, the expression of inflammatory cytokines manifested no significant difference. Although it has been previously demonstrated that MSCs directly regulate the proliferation and differentiation of CD4^+^ T cells in vitro [53], which cannot well reproduce the real interaction between MSCs and host in vivo due to the existence of the complicated physiological process influenced by various factors, as indicated by our results that the effect on restoring Treg/Th2/Th17 balance by HUMSCs in colitis disappeared following gut microbiota depletion. Subsequent FMT experiments were performed to furtherly verify the microbiota-dependent mechanism. In consistent with that of MSC itself, FMT(MSC) alleviated symptoms of colitis profoundly better than FMT(DSS), as well the reduced inflammatory responses and the promoted Treg/Th2/Th17 cells homeostasis restoration. Based on these results, we demonstrated that HUMSCs-mediated gut microbiota played a dominant role in alleviating the colonic immune inflammatory response in vivo.

Although the cooperation between gut microbiota and host immunity has been demonstrated, the mechanism by which the gut microbiota regulates T cells remains unclear. Multitudes of studies have elucidated that SCFAs as key metabolites derived from gut microbiota, participate in the maintenance of intestinal homeostasis as well as in the pathogenesis of IBD [12]. The principal SCFAs, including acetate, propionate and butyrate, are distinctly reduced in the feces of IBD patients, and some individuals with IBD have benefited from butyrate enemas [54]. Here, following the changes in the gut microbiota by HUMSCs, we observed that feces from MSCs-treated mice had increased levels of the SCFAs, especially acetate, propionate and butyrate compared to untreated colitis mice, compatible with the increased abundance of *Akkermansia, Clostridia_UCG_014, Faecalibaculum*, *Blautia*, *Anaerotruncus*, and *Lachnospiraceae_NK4A136_group.* Correlation analysis revealed that six differential bacterial genera enriched in MSCs-treated group showed positive correlation with SCFAs. Particularly, the genera *Clostridia_UCG_014* had significant positive correlation with various SCFAs. *Akkermansia* displayed a strong positive correlation with butyrate, and *Faecalibaculum* showed obvious positive correlation with acetate and butyrate. However, the bacteria *Lactobacillus* and *Alloprevotella* enriched in the colitis group manifested negative correlation with SCFAs, although the statistical differences were not significant. Notably, the transfer of feces from MSCs-treated mice significantly promoted the production of SCFAs compared to the transfer from only DSS-treated mice, indicating that the release of SCFAs depended on SCFAs-producing bacteria. SCFAs have been confirmed to suppress the proliferation activity of CD4^+^ T cells, significantly promote Treg differentiation, and upregulate the secretion of IL-10, improving colon inflammation [10]. Consistent with these data, we also verified that SCFAs supplementation could significantly restore colonic Treg/Th2/Th17 balance, regulate cytokine secretion, and suppress colonic inflammatory responses. As expected, the gavage of SFF from MSCs-treated mice promoted the recovery of colonic immune inflammation and the elevations of acetate, propionate, and butyrate with the similar trend as FMT(MSC) or MSC itself, when compared to SFF(DSS) group mice. Notably, either SCFAs or SFF gavage primarily affected colonic LP T cell responses, but not MLN. Which might because of the sterility in SFF and the gut microbiota also exhausted by ABX, bacterial translocation effect or bacteria-related mechanisms disappeared. Besides, SCFAs were mainly absorbed by intestinal epithelial cells [55], thus, local mucosal immune regulation might predominate as previously reported [11]. Additionally, to furtherly confirm the regulatory role of SCFAs on immune inflammatory response, the correlation between SCFAs and cytokines was analyzed. With similarity to previous study [41], no matter in DSS/MSC, FMT or SFF model system, SCFAs were inversely correlated with pro-inflammatory factors and positively with anti-inflammatory factors, and especially in FMT and SFF model systems, the correlation was more significant. The data supported a mechanism whereby HUMSCs caused increased abundance of SCFAs-producing bacteria, resulting in increased acetate, propionate, and butyrate, which in turn mediated the suppression of colitis via restoring T cells immune homeostasis.

This study is the first to explore the role of gut microbiome in mediating the protective effect of MSCs on colitis. However, it should also be acknowledged that it is lack of in-depth mechanism investigation in the present study, such as the mechanism of SCFAs regulating CD4^+^T homeostasis. Although previous studies have reported SCFAs can regulate the proliferation and differentiation of T cells by binding to their corresponding receptors [11], the specific receptor and its downstream signaling pathway are worthy of further exploration. In addition, our previous findings suggest that MSCs remodeled the structure and diversity of gut microbiota may partially by regulating the Tregs-lgA response, influencing intestinal lgA secretion [22], and while the other mechanisms are still need to be clarified, for example whether MSCs or their secreted components can be taken up by the intestinal microbiota furtherly affecting the community of gut flora.

## Conclusions

**Fig. 9:**
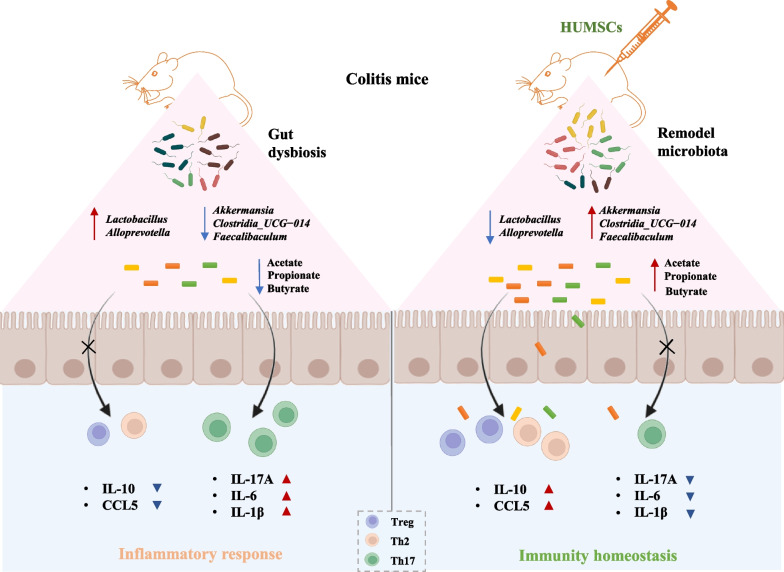
The delivery of HUMSCs remodeled the composition and diversity of gut flora, especially up-regulated SCFAs-producing bacterial abundance, such as *Akkermansia*, *Faecalibaculum*, and *Clostridia_UCG_014*, subsequent increased the output of acetate, propionate, and butyrate, which in turn promoted a pronounced T cells homeostasis-restoring and anti-inflammatory, resulting in the amelioration of intestinal injury

The present study advances understanding of HUMSCs in the protective effects on colitis, providing evidence for the essential role of gut microbiota in the recovery of colonic inflammation by HUMSCs. As our data showed, the delivery of HUMSCs remodeled the composition and diversity of gut flora, especially up-regulated SCFAs-producing bacterial abundance, such as *Akkermansia*, *Faecalibaculum*, and *Clostridia_UCG_014*, subsequent increased the output of acetate, propionate, and butyrate, which in turn promoted a pronounced T cells homeostasis-restoring and anti-inflammatory, resulting in the amelioration of intestinal injury (Fig. 9). Collectively, HUMSCs ameliorated colon inflammation in a gut microbiota-dependent manner, and the underlying protective mechanism was associated with the improved Treg/Th2/Th17 balance in intestinal mucosa mediated via the increased microbiota-derived SCFAs production. Therefore, the microbiome-metabolite-immune axis provides a novel role for MSCs as a potential gut microbiota modulator to treat IBD.

### Supplementary Information

**Additional file 1**. **Fig. S1.** The absolute numbers of Treg/Th2/Th17/Th1 cells in LP and MLN with HUMSCs administration. **Fig. S2.** HUMSCs treatment had no effect on Treg/Th2/Th17 balance in spleen. **Fig. S3–5.** HUMSCs treatment regulated the abundance, diversity and composition of gut microbiota in colitis mice. **Fig. S6** The absolute numbers of Treg/Th2/Th17 cells in LP and MLN in ABX-colitis mice. **Fig. S7.** The absolute numbers of Treg/Th2/Th17 cells in LP and MLN in FMT-colitis mice. **Fig. S8.** SCFAs standards total ions chromatogram (TIC) chart. **Fig. S9.** The absolute numbers of Treg/Th2/Th17 cells in LP and MLN in SFF-colitis mice.

**Additional file 2**. The original Ct value data of the target genes in cytokine microarray.

## Data Availability

The data that support the findings of this study are available from the corresponding author upon reasonable request, and the dataset presented in this study is available in the NCBI Sequence Read Archive (SRA) repository under accession number PRJNA885240 (https://dataview.ncbi.nlm.nih.gov/object/PRJNA885240).

## Abbreviations

IBD: Inflammatory bowel disease
MSC: Mesenchymal stem cell
DSS: Dextran sodium sulfate
HUMSC: Human umbilical cord MSC
SCFA: Short-chain fatty acid
ABX: Antibiotic cocktail
FMT: Fecal microbiota transplantation
SFF: Sterile fecal filtrate
ELISA: Enzyme-linked immunosorbent assay
LP: Lamina propria
MLN: Mesenteric lymph node
IL-17A: Interleukin-17A
IL-1β: Interleukin-1β
IL-6: Interleukin-6
IL-10: Interleukin-10
CCL5: C–C chemokine ligand 5
IL-4: Interleukin-4
Treg: Regulatory T
Th2: T helper type 2
Th17: T helper type 17
Th1: T helper type 1
CD: Crohn’s disease
UC: Ulcerative colitis
CAC: Colitis-associated colorectal cancer
PBS: Phosphate-buffered saline
DAI: Disease Activity Index
H&E: Hematoxylin and eosin
PCoA: Principal coordinate analysis
NMDS: Non-Metric Multi-Dimensional Scaling
