# Supplementary material for: Human umbilical cord mesenchymal stem cells ameliorate colon inflammation via modulation of gut microbiota-SCFAs-immune axis
Source: Stem Cell Res Ther. 2023 Sep 25;14:271. doi: 10.1186/s13287-023-03471-9 (PMC10521524; doi:10.1186/s13287-023-03471-9)
Supplement: Supplementary file 1 — Additional file 1. Fig. S1. The absolute numbers of Treg/Th2/Th17/Th1 cells in LP and MLN with HUMSCs administration. Fig. S2. HUMSCs treatment had no effect on Treg/Th2/Th17 balance in spleen. Fig. S3–5. HUMSCs treatment regulated the abundance, diversity and composition of gut microbiota in colitis mice. Fig. S6 The absolute numbers of Treg/Th2/Th17 cells in LP and MLN in ABX-colitis mice. Fig. S7. The absolute numbers of Treg/Th2/Th17 cells in LP and MLN in FMT-colitis mice. Fig. S8. SCFAs standards total ions chromatogram (TIC) chart. Fig. S9. The absolute numbers of Treg/Th2/Th17 cells in LP and MLN in SFF-colitis mice. [file 13287_2023_3471_MOESM1_ESM.docx]

**Fig. S1** HUMSCs treatment reshaped Treg/Th2/Th17 balance in colitis mice. (a) The bar charts of the absolute numbers of Treg, Th2, Th17, and Th1 cells between the DSS/PBS and DSS/MSC groups were displayed in colonic LP. (b) The bar charts of the absolute numbers of Treg, Th2, Th17, and Th1 cells between the DSS/PBS and DSS/MSC groups were displayed in MLN. n=5 per group. Data were presented as means ± SD. *P* values were calculated using Unpaired T-test, * *p* < 0.05, ** *p* <0.01, NS indicates *p* >0.05.

**Fig. S2** HUMSCs treatment had no effect on Treg/Th2/Th17 balance in spleen. Treg, Th2, Th17, and Th1 cells in spleen from Control, DSS/PBS and DSS/MSC groups were analyzed by flow cytometry and bar charts of the percentages and the absolute numbers were displayed. n=5 per group. Data were presented as means ± SD. Comparisons of parameters for three groups were made by ANOVA followed by Tukey's test for three groups, and *P* values were calculated using an unpaired two-tailed Student’s *t*-test for two groups. NS indicates *p* >0.05.

**Fig. S3** HUMSCs treatment regulated the abundance of gut microbiota in colitis mice. Histograms of relative abundance for top 10 microbes at the class (a) and order (b) levels. (c) The abundance changes of *Bacteroidota*, *Proteobacteria*, and *Firmicutes* among Control, DSS/PBS, and DSS/MSC groups (n=6). Data were presented as means ± SD. *P* values were calculated by ANOVA followed by Tukey‘s test, * *p* < 0.05. (d) The abundance changes of *Muribaculaceae*, *Bacteroidaceae* and *Lachnospiraceae* among Control, DSS/PBS, and DSS/MSC groups (n=6). Data were presented as means ± SD. (e) The abundance changes in genus between DSS/PBS and DSS/MSC groups, and Control and DSS/MSC groups. Data were presented as means ± SD. *P* values were calculated using Unpaired T-test.

**Fig. S4** HUMSCs treatment regulated the diversity of gut microbiota in colitis mice. (a) Alpha diversity boxplot of observed_otus. (b) Alpha diversity boxplot of simpson index.

**Fig. S5** HUMSCs treatment regulated the composition of gut microbiota in colitis mice. (a) The alterations of genera *Lactobacillus* and *Alloprevotella* among Control, DSS/PBS and DSS/MSC groups based on all-against-all algorithm of LDA coupled with LEfSe. (b) The alterations of genera *Akkermansia* and *Clostridia_UCG_014* among Control, DSS/PBS and DSS/MSC groups based on all-against-all algorithm of LDA coupled with LEfSe.

**Fig. S6** The effect on restoration of Treg/Th2/Th17 balance by HUMSCs administration in ABX-colitis mice was abrogated by antibiotic treatment. (a) The absolute numbers of Treg, Th2 and Th17 cells in colonic LP between ABX/DSS/PBS and ABX/DSS/MSC groups. (b) The absolute numbers of Treg, Th2 and Th17 cells in MLN between ABX/DSS/PBS and ABX/DSS/MSC groups. n=5 per group. Data were presented as means ± SD. *P* values were calculated using Unpaired T-test, NS indicates *p* >0.05.

**Fig. S7** The balance of Treg/Th2/Th17 in ABX-colitis mice was restored by FMT from HUMSCs-treated mice. (a) The absolute numbers of Treg, Th2 and Th17 cells in colonic LP among FMT(Control), FMT(DSS) and FMT(MSC) groups. (b) The absolute numbers of Treg, Th2 and Th17 cells in MLN among FMT(Control), FMT(DSS) and FMT(MSC) groups. n=5 per group. Data were presented as means ± SD. *P* values were calculated by ANOVA followed by Tukey's test, * *p* < 0.05, ** *p* <0.01, *** *p* < 0.001, NS indicates *p* >0.05.

**Fig. S8** Short-chain fatty acid (SCFAs) standards total ions chromatogram (TIC) chart.

**Fig. S9** The balance of Treg/Th2/Th17 in ABX-colitis mice was restored by SFF from HUMSCs-treated mice. (a) The absolute numbers of Treg, Th2, and Th17 cells in colonic LP among SFF(DSS), SFF(MSC) and SCFAs groups. (b) The absolute numbers of Treg, Th2, and Th17 cells in MLN among SFF(DSS), SFF(MSC) and SCFAs groups. n=5 per group. Data were presented as means ± SD. *P* values were calculated by ANOVA followed by Tukey's test, * *p* < 0.05, ** *p* <0.01, *** *p* < 0.001, NS indicates *p* >0.05.
